# Development of a patient-centred electronic review template to support self-management in primary care: a mixed-methods study

**DOI:** 10.3399/BJGPO.2022.0165

**Published:** 2023-05-31

**Authors:** Kirstie McClatchey, Aimee Sheldon, Liz Steed, Jessica Sheringham, Francis Appiagyei, David Price, Vicky Hammersley, Stephanie Taylor, Hilary Pinnock

**Affiliations:** 1 Asthma UK Centre for Applied Research, Usher Institute, The University of Edinburgh, Scotland, Edinburgh, UK; 2 Institute for Population Health Sciences, Barts and the London School of Medicine and Dentistry, Queen Mary University, London, UK; 3 Department of Applied Health Research, University College London, London, UK; 4 Optimum Patient Care, Cambridge, UK; 5 Observational and Pragmatic Research Institute, Singapore, Singapore; 6 Centre of Academic Primary Care, Division of Applied Health Sciences, University of Aberdeen, Aberdeen, UK

**Keywords:** asthma, self-management, primary health care, general practice

## Abstract

**Background:**

Electronic templates are frequently used in long-term condition (LTC) reviews (for example, asthma) to act as reminders and improve documentation; however, they can restrict patient-centred care and opportunities for patients to discuss concerns and self-management.

**Aim:**

The IMPlementing IMProved Asthma self-management as RouTine (IMP^2^ART) programme aimed to develop a patient-centred asthma review template that encourages supported self-management.

**Design & setting:**

This was a mixed-methods study, which integrated qualitative and systematic review data, primary care Professional Advisory Group feedback, and qualitative data from clinician interviews.

**Method:**

Aligned with the Medical Research Council complex intervention framework, a template was developed in the following three phases: (1) development phase, which consisted of a qualitative exploration with clinicians and patients, a systematic review, and prototype template development; (2) feasibility pilot phase, which involved feedback from clinicians (*n* = 7); and (3) pre-piloting phase, which consisted of delivering the template within the IMP^2^ART implementation strategy (incorporating the template with patient and professional resources) and eliciting clinician feedback (*n* = 6).

**Results:**

Template development was guided by the preliminary qualitative work and the systematic review. A prototype template was developed with an opening question to establish patient agendas, and a closing prompt to confirm agendas have been addressed and an asthma action plan provided. The feasibility pilot identified refinements needed, including focusing the opening question on asthma. Pre-piloting ensured integration with the IMP^2^ART strategy.

**Conclusion:**

Following the multi-stage development process, the implementation strategy, including the asthma review template, is now being tested in a cluster randomised controlled trial.

## How this fits in

Electronic templates are often used in LTC reviews (for example, asthma). They can act as reminders and improve documentation of key measures. However, templates have also been found to restrict patient-centred care and opportunities for patients to discuss concerns and self-management. As part of a programme of work aiming to develop and evaluate a strategy to improve implementation of supported asthma self-management in primary care (IMP^2^ART), a patient-centred asthma review template was developed to encourage supported self-management. The defining elements of the IMP^2^ART template are that it includes an opening question to establish patient agendas, and a closing prompt to verify the agenda has been addressed and an asthma action plan has been provided. These key elements encourage patient-centred care, by prompting clinicians to work collaboratively with patients, and facilitating asthma action plan provision. For clinicians, the findings can inform the development of patient-centred electronic review templates, addressing some of the recognised disadvantages of template-structured care.

## Introduction

LTCs account for approximately 15 million premature deaths globally each year,^1^ emphasising a need to improve care. Electronic disease templates are commonly used in healthcare systems to structure LTC management and data recording.^2^ The data retrieved via review templates can be used to enable pay-for-performance, which, although it has not been shown to change mortality,^3^ can impact positively on healthcare quality.^4^ In the UK, trained nurses usually conduct focused LTC reviews using electronic templates, often discussing lifestyle issues and changes to disease management.^2^ Review templates can be valuable resources, improving the documentation of key measures.^5^ However, templates may encourage a checklist approach to LTC care;^6^ prioritise the healthcare professional agenda over the patient's;^7^ and limit opportunities for patients to discuss self-management.^6^ Patient-centred care involves healthcare professionals working collaboratively with patients to support them to develop the knowledge, skills, and confidence they need to self-manage more effectively and make informed decisions about their health and health care.^8^ In order to support LTC care and self-management, templates should be designed with patient-centred care in mind.

Asthma is an LTC that affects approximately 5.4 million people in the UK.^9^ Supported self-management for asthma, which includes patient education, regular review, and personalised asthma action plan provision, has been recommended by guidelines for 30 years.^10^ A recent meta-review found that supported asthma self-management can reduce hospitalisations, accident and emergency attendances, and unscheduled care consultations.^11^ Despite the evidence, it is poorly implemented in practice. Only half (52%) of the responders to a survey conducted by Asthma UK (whose self-management resources are widely promoted)^12^ owned an asthma action plan;^9^ in the authors' review of clinical records, only 6% of people with asthma had a record of being provided with an action plan.^13^

In response to the poor implementation of supported asthma self-management, the IMP^2^ART programme was developed. IMP^2^ART aims to develop and evaluate a three-level implementation strategy for primary care comprising resources for patients, professional training, and organisational support (including an asthma review template). This article reports the design and development, within the context of the IMP^2^ART research, of a theoretically-informed, patient-centred electronic asthma review template, which aims to encourage self-management and asthma action plan ownership.

## Method

**Figure 1.**
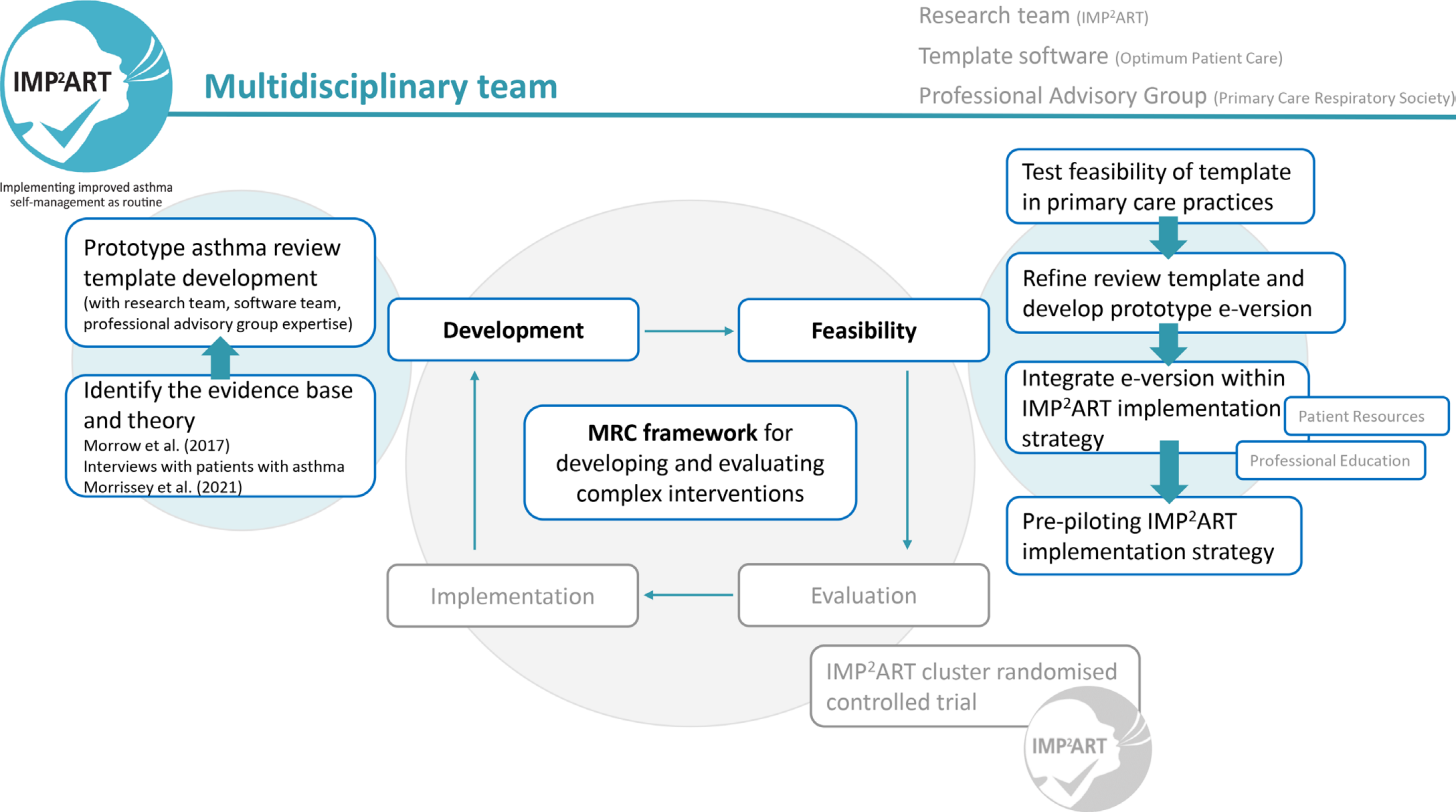
Asthma review template development phases aligned with the Medical Research Council (MRC) framework for developing and evaluating complex interventions

The programme of work aligns with the development and feasibility stages of the Medical Research Council (MRC) framework for developing and evaluating complex interventions,^14^ guidance on developing complex interventions to improve health and health care,^15^ and follows the guidance for reporting intervention development studies in health research (GUIDED).^16^ The IMP^2^ART research team consists of academics and healthcare professionals (nurses, GPs, health psychologists) based within the Asthma UK Centre for Applied Research (AUKCAR). In addition, Optimum Patient Care (OPC), a not-for-profit social enterprise improving the diagnosis, treatment, and management of chronic diseases within primary care, assisted with building the asthma review template. A Professional Advisory Group of GPs and nurses (*n* = 10) was established from the Primary Care Respiratory Society (PCRS) to advise on template content. They met twice (by video-conference) during the development and discussed template design and patient-centredness. Figure 1 displays the overarching asthma review template development phases, which aligned to the MRC framework for developing and assessing feasibility of complex interventions.^14^

### MRC framework

#### Development phase

Preliminary work included a qualitative exploration of supporting asthma self-management in UK primary care with general practice staff,^17^ and interviews with UK patients living with asthma to explore views about routines and resources used to support care (both current and aspirational). Additionally, a systematic review was conducted to investigate the impact of using LTC review templates during consultations.^5^ Building on the expertise of the research team, OPC, and the Professional Advisory Group, the explorative and review work was followed by the development of a system-agnostic prototype IMP^2^ART asthma review template (in PDF format; see Figure 1).

#### Feasibility pilot phase

Clinical staff were recruited from five UK general practices, and they were provided with the IMP^2^ART pdf-prototype asthma review template for feedback (Figure 1). Semi-structured qualitative interviews were used to explore their thoughts and recommendations for the template. The interviews were carried out by AS and KM between February and May 2019, and were audio-recorded, transcribed verbatim, and analysed in NVivo (version 11) using framework analysis.^18^

#### Pre-piloting the IMP^2^ART asthma review template within the IMP^2^ART implementation strategy

Following refinement, an electronic prototype version of the template (for SystmOne and EMIS) was integrated with patient resources and professional training components of the IMP^2^ART strategy for pre-piloting in four general practices (Figure 1). A sample of practice staff from each of the four pre-pilot practices were interviewed by KM between October and November 2019 using semi-structured interviews to explore experiences of using the IMP^2^ART strategy (including the template). Interviews were audio-recorded, transcribed verbatim, and analysed using framework analysis^18^ in NVivo (version 11). The refined asthma review template was then incorporated into the IMP^2^ART implementation strategy for evaluation in a cluster randomised controlled trial RCT.

## Results

Overarching results can be found in Supplementary Table S1.

### Development phase

#### Qualitative exploration

The preliminary qualitative exploration with 33 general practice staff (23 GPs, seven nurses, three administrative staff) found that annual asthma reviews and supporting self-management was largely a nurse-led task, and reviews were mostly structured around electronic templates.^17^ In addition to describing templates as an aide memoire, clinicians considered that templates had the potential to promote and facilitate supported self-management, including provision of asthma action plans. Poor integration with IT systems, alert fatigue, duplication of effort, and too many tick boxes were identified as barriers.

Interviews with patients living with asthma (*n* = 10 [seven male, three female]) across four UK general practices) found that most were aware that their clinician used the computer, or some form of template on the computer, during their review. However, this did not restrict the review process, as most felt able to discuss any issues they had about their asthma, and they felt involved in the decisions that were made regarding their asthma.

#### Systematic review

The IMP^2^ART systematic review concluded that templates can improve documentation of key measures, and act as a reminder tool during consultations; however, concerns were raised that templates may act as a barrier to providing patient-centred care^5^ by restricting the review process and prioritising the healthcare professional's agenda over the patient’s.^5^ In line with the findings by Morrow *et al*,^17^ the review recommended that templates should incorporate free-text options for documentation (as well as coded items), and should include education and self-management items to prompt healthcare professionals to encourage and support patient self-management practices. A specific suggestion to improve patient-centredness was that templates should begin with an opening question to establish the patient’s agenda, and also a closing question to confirm concerns have been addressed.

#### Prototype IMP^2^ART asthma review template development

The PCRS Professional Advisory Group welcomed the patient-centred and shared-decision making approach to the template, and specifically highlighted that the generic opening question about the patient’s agenda (‘*What is the patient’s agenda?’*) should focus on asthma reviews (*'Thank you for coming to an asthma review, is there anything in particular you would like to talk about?'*). Utilising the evidence base, following professional advice, and including the necessary Quality and Outcomes Framework (QOF)^19^ components, a prototype IMP^2^ART asthma review template was produced by OPC.

### Feasibility pilot phase

In total, seven clinical staff (four nurses, two GPs, and one pharmacist; all female) from five general practices participated in interviews discussing the prototype IMP^2^ART template. Five themes were identified and are detailed in Supplementary Table S1 and below.

#### Helpful components

Clinicians found a range of the IMP^2^ART template features helpful. These included the following: electronic health record auto-populated information; links to information for patients; the dedicated section for assessment of uncontrolled asthma; user-friendliness; patient-centred questions; and the closing summary of management recommendations. For less experienced staff, a prompt to ensure the review was complete was found to be helpful.

#### Content, design, and timing

Clinicians thought the IMP^2^ART template covered the pertinent aspects of a review. Both GPs thought the template was long, with one commenting on several items she was unlikely to use. The nurses and pharmacist, however, thought the amount of content was as expected, with some implication that more content was better than less:

*'There’s a lot to work with … you’ve covered most bases that I would see anyway.'* (Nurse 2, female)

Clinicians welcomed the addition of template-embedded patient information links, although it was noted they should be used within the context of a range of different resources for different patients. All interviewees considered that the template appeared to be simple to navigate. Despite this, the volume of questions initially seemed daunting (this was because the prototype template was printed in a single screen without visualisation of discrete tabs). Once clinicians explored the content further, they concluded the template fitted with their current review sequence; it would be user-friendly as there was less complex navigation, and fields were optional. Clinicians had mixed opinions on whether the template was achievable in time-limited reviews. Several thought it was appropriate for a 20-minute consultation but others expressed some concerns about the time required for completion. Clinicians observed that timing is often patient or practice-dependent:

*'So although I initially said "oh, it’s quite long," you’re not going to be asking your well-controlled asthmatics all of these questions.'* (GP 2, female)

#### Patient-centredness

The IMP^2^ART template was viewed as patient-centred. Clinicians regarded the opening agenda question, *'Thank you for coming to an asthma review, is there anything in particular you would like to talk about?'*, positively. Several expressed that it facilitated holistic care by concentrating on what the patient wants to address, helping divert focus from the clinician, and engaging the patient by making them feel as if they were gaining something from the review. Clinicians thought this cue-based approach had the potential to make patients more open to education:

*'What’s the thing YOU want to talk about ... that’s absolutely what we’re interested in in our practice is trying to have a holistic review … focusing on the patient’s agenda first … it’s hard to engage them in this so I think that’s a useful opening line.*' (GP 2, female)

#### Implementing into practice

All clinicians said they would be comfortable with implementing the IMP^2^ART template in their practice. Reasons for comfort with the template included similarity to their current template and compliance with the QOF, which determined performance-related payments:^19^

*'I don’t think it’s dissimilar to what we already use so I don’t see the issue there.*' (GP 1, female)

#### Recommendations

Three clinicians highlighted that the opening agenda question needed to be open enough to allow the patient to express their concerns, but should avoid shifting the focus of the consultation from asthma:

*'You want it to be open but you’re wanting to be focused on their asthma rather than whether they got parked or not.*' (GP 1, female)

Four clinicians recommended changes to template content including the addition of missing content and removal of superfluous items. Suggested additional content included inhaled corticosteroid adherence and/or dose, spacer use, and replacement. More detail on asthma triggers was regarded as important to note, owing to their possible impact on quality of life.

The proposed removals were as follows: age of asthma onset; eosinophil count; spirometry; smoking questions (shorten to fewer questions); and use of the asthma control test (ACT)^20^ as opposed to the Royal College of Physicians three questions (RCP3Q)^21^ or Global Initiative For Asthma (GINA) assessment of asthma control.^22^ Recommendations for IT use included the following: software-compatible templates; the ability to print off the link to the patient-facing website (or a code that could be scanned); and attachment of completed action plans to the electronic health record for re-evaluation at subsequent reviews.

### Refining after the feasibility pilot interviews

Following feedback from the clinicians, the multidisciplinary team had a consensus discussion and made refinements to the template, which included reducing the number of questions about smoking, and changing the template opening question to be more asthma focused (*‘What would you like to discuss about your asthma?’*).

### Pre-piloting the electronic IMP^2^ART asthma review template as a component of the IMP^2^ART implementation strategy

Six clinical staff (four GPs [two male, two female], and two nurses [female]) across four general practices participated in interviews following template refinements. The electronic prototype version was integrated with the IMP^2^ART implementation strategy (patient resources and professional training) in the pre-pilot. Two participants provided additional feedback via email after the interviews had been completed. Three themes were identified and are detailed below, and in Supplementary Table S1.

#### Perceptions of the template

Clinicians were positive about the IMP^2^ART template available on their systems, and considered that it covered what was expected:

*'… it is actually quite comprehensive.*' (Nurse 1, female)

Participants welcomed the patient-centred focus of the template, in particular the opening question:

*'… I think it’s good … because obviously we’re trying to create that holistic care.*' (GP 2, male)

#### Template implementation

Participants indicated that they had implemented, or planned to implement, the IMP^2^ART template into their care delivery:

*'We have spoken to everyone and started to use the template now because it has a lot more information there than what was given in our original template …*' (GP 2, male)

#### Suggested changes to the template

Participants made a number of detailed suggestions about items they would like to be included, revised, or omitted (see Supplementary Table S1), and one participant asked if the personalised asthma action plan could be saved to the record for future reference.

### Refining after the pre-pilot interviews

Refinements, such as the addition of some suggested tick boxes, were added to the IMP^2^ART template following the interviews. The PCRS Professional Advisory Group had no further comments or suggestions for the template. The template was then formatted for different clinical systems (EMIS, SystmOne, and Vision) for future use in the IMP^2^ART cluster RCT.

### A dynamic and iterative process

The process of developing the finalised IMP^2^ART asthma review template followed key principles of intervention development that it was dynamic, iterative, creative, and open to change.^15^ The template not only evolved during development, feasibility, and pre-piloting phases, but also continued to evolve in accordance with changing contexts. For example, following the COVID-19 pandemic in 2020, the PCRS Professional Advisory Group were consulted to discuss remote consulting and considered if any further changes to the template were needed (they decided not). Further, the electronic prototype version of the template was developed for pre-piloting using Read codes. In England, SNOMED Clinical Teams (CT) codes were adopted in 2020,^23^ thus the template was adapted to include SNOMED CT codes before the start of the cluster RCT in early 2021. Finally, as the IMP^2^ART asthma review template was designed as part of an implementation strategy, practices are able to adapt the template to add or remove fields to suit individual practice routines, which will be monitored as part of the implementation process.

## Discussion

### Summary

The current article has described the development of an asthma review template within a research programme. Aligned to the MRC framework,^14^ the process included a development phase drawing on existing literature, a feasibility phase to explore clinician views of the review template, and a real-world pre-pilot of the review template as a component of the IMP^2^ART implementation strategy. Following the feasibility and pre-pilot testing of the review template, feedback was considered and changes were made. The defining elements of the IMP^2^ART template include an opening question to establish patient agendas, and a closing prompt to verify the agenda has been addressed and an asthma action plan has been provided. These key elements were perceived as encouraging patient-centred care, by prompting clinicians to work collaboratively with patients, and facilitating asthma action plan provision.

### Strengths and limitations

A major strength of the research was the cross-discipline work underpinning the development of the asthma review template. Development, feasibility testing, and pre-piloting with patients and clinicians currently working in general practice further strengthened the work and its applicability to real-world practice. Additionally, the testing phases allowed for refining of the review template in an iterative and ongoing process. Limitations of the research included small sample sizes for qualitative interviews, and the lack of patient interviews to explore their thoughts of the newly developed template, which may have provided additional insight to the patient-centred focus of the template.

### Comparison with existing literature

Understanding and adapting to context is crucial in implementation research.^24^ The initial qualitative exploration, ongoing advice from a primary care stakeholder panel, as well as the iterative testing in routine practice, ensured the template fits (and if necessary can be further adapted to) the routines of UK primary care. The core principles of ensuring patient-centredness and alignment with guidelines, however, remain generalisable and pertinent to any setting providing proactive care for people with LTCs.

The findings of the current research regarding the opening template question addressing the patient’s agenda positively aligned with prior research. For example, a study evaluating a patient-centred template that began with an opening question about the patient's most important concerns, found that clinicians highly valued the enquiry about the patient’s agenda, and it allowed for the identification of unmet health needs through the patients revealing previously unmentioned symptoms.^2^

### Implications for research and practice

The authors conclude that a multi-stage development process, aligned with the MRC complex intervention framework, contributed to the design and delivery of the patient-centred asthma review template. The IMP^2^ART strategy (incorporating the review template, as well as patient resources and professional education) is now being tested in a UK-wide cluster RCT (ISRCTN reference number: ISRCTN15448074 ) that is evaluating implementation (action plan ownership) and health outcomes (unscheduled care). For clinicians, the findings can inform the development of patient-centred electronic review templates, addressing some of the recognised disadvantages of template-structured care.

## Data availability:

The data that support the findings of this study are available from the corresponding author upon reasonable request.

## Data

The data that support the findings of this study are available from the corresponding author upon reasonable request.
